# Structural Characterization of a Proto-Type Galectin from *Cinachyrella* sp. and Evaluation of Its Selective Bacterial Glycan Recognition and Antibiofilm Activity

**DOI:** 10.3390/microorganisms14071442

**Published:** 2026-06-30

**Authors:** Juliana Sampaio Nogueira Marques, Francisco Regivânio Nascimento Andrade, Renato Cézar Farias Torres, Israel Ferreira Barbosa Junior, Gloria Steffanne Damasio da Silva, Renata Pinheiro Chaves, Ellen Araújo Malveira, Elielton Nascimento, Ulisses Pinheiro, Mayron Alves de Vasconcelos, Edson Holanda Texeira, Rômulo Farias Carneiro, Celso Shiniti Nagano, Alexandre Holanda Sampaio

**Affiliations:** 1 Marine Biotechnology Laboratory—BioMar-Lab, Department of Fisheries Engineering, Federal University of Ceará, Pici Campus, Block 871, Fortaleza 60440-970, CE, Brazil; julyanasampaio@hotmail.com (J.S.N.M.); regi.andrade.biotec@gmail.com (F.R.N.A.); renatocft@gmail.com (R.C.F.T.); israeljunior45512@gmail.com (I.F.B.J.); gloria.damasio@alu.ufc.br (G.S.D.d.S.); renatapinheirochaves@gmail.com (R.P.C.); naganocs@ufc.br (C.S.N.); 2 Integrated Biomolecules Laboratory—LIBS, Department of Pathology and Legal Medicine, Federal University of Ceará, Monsenhor Furtado Street, s/n, Fortaleza 60430-160, CE, Brazil; ellenmalveira11@gmail.com (E.A.M.); mayronvasconcelos@gmail.com (M.A.d.V.); edson@ufc.br (E.H.T.); 3 Laboratório de Poriferos, Departamento de Zoologia, UFPE—Universidade Federal de Pernambuco, Av. Prof Moraes Rego, 1235, Cidade Universitária, Recife 50670-901, PE, Brazil; biologoefn@gmail.com (E.N.); uspinheiro@hotmail.com (U.P.); 4 Faculty of Education of Itapipoca (Facedi), State University of Ceará, Av. da Universidade, s/n, Itapipoca 62500-000, CE, Brazil

**Keywords:** lectin, bacterial biofilm, marine bioprospecting, marine sponges

## Abstract

Marine sponges represent a rich source of lectins with diverse biological activities and biotechnological potential. In this study, we report the purification and comprehensive biochemical and structural characterization of a lectin (CspL) from the marine sponge *Cinachyrella* sp. and evaluate its effects on bacterial agglutination, planktonic bacterial growth, and biofilm formation. CspL was isolated using classical chromatographic approaches and identified as a galectin-like protein based on sequence similarity, conserved carbohydrate-recognition motifs, and a predominance of β-sheet structures revealed by circular dichroism. Oligomeric analysis indicated a homotetrameric organization, consistent with the quaternary structure described for other sponge proto-type galectins. Carbohydrate-binding assays demonstrated that CspL preferentially recognizes galactoside-containing motifs, showing strong inhibition by mucin-type glycoproteins, while displaying lower affinity toward more complex glycan structures. This binding profile suggests a preference for accessible carbohydrate epitopes, likely associated with its canonical galectin architecture. Regarding antibacterial activity, CspL also exhibited selective, carbohydrate-dependent bacterial agglutination, particularly against *Staphylococcus aureus* strains. In addition, CspL exhibited antibiofilm activity against *S. aureus* and *Escherichia coli*, significantly reducing biofilm biomass and viable cell counts. Additionally, the lectin modulated antibiotic activity, showing synergistic effects with tetracycline and strain-dependent interactions with oxacillin. Together, these findings highlight CspL as a structurally conserved yet functionally relevant member of sponge galectins and reinforce the role of structural diversity in shaping glycan recognition and antimicrobial activity in marine lectins.

## 1. Introduction

Lectins represent a class of proteins capable of binding specifically and reversibly to carbohydrates, recognizing glycoconjugates, glycolipids, oligosaccharides, monosaccharides, and proteoglycans. Although they may play important roles in the innate immune system of animals, they do not belong to any class of immunoglobulins [1].

Galectins are a family of lectins characterized by their ability to recognize and bind to galactosides through a carbohydrate-recognition domain (CRD) containing highly conserved amino acid residues. Galectins can function as pattern recognition proteins (PRPs) by identifying pathogen-associated molecular patterns (PAMPs) [2].

Some sponge-derived galectins have demonstrated significant biotechnological potential due to their ability to recognize pathogenic bacteria as well as tumoral cells and therefore represent promising candidates for biotechnological applications [3,4]. For instance, ALL (*Aplysina lactuca* lectin) and CCL (*Chondrilla caribensis* lectin) have been shown to reduce biofilm biomass and to agglutinate bacterial strains such as *S*. *aureus*, *S*. *epidermidis*, and *E*. *coli*. In addition, ALL exhibited a synergistic effect when combined with ampicillin and tetracycline against *S*. *epidermidis*, whereas CCL has also been reported to display leishmanicidal activity [5,6].

Sponge-derived galectins have been increasingly structurally characterized in recent years, revealing a notable diversity in their architectural features. A subset of these lectins, including ALL, AcrL (*Aiolochroia crassa* lectin), and CCL, exhibit additional structural elements, such as an extended loop proximal to the CRD [5,6,7]. In contrast, the only crystallographically resolved sponge galectin to date, derived from *Cinachyrella* sp., displays the canonical β-sandwich fold typical of proto-type galectins, with conserved CRD architecture [8].

In light of these structural considerations, further bioprospecting efforts are required to better understand the evolutionary diversification of galectin architecture in basal metazoans. In this context, the genus *Cinachyrella* emerges as a particularly relevant model, as it comprises lectins with demonstrated biological activities but limited structural characterization. For instance, a lactose-binding lectin of approximately 15 kDa isolated from *Cinachyrella alloclada* exhibited anti-HIV activity, while a similar lectin from *Cinachyrella apion*, also around 15 kDa and lactose-binding, demonstrated leishmanicidal and antitumoral effects [9,10,11]. In addition, galectins isolated from *Cinachyrella* sp. (specimens collected in Okinawa, Japan) have also been reported to modulate ionotropic glutamate receptors in the central nervous system [8,12].

Therefore, this study aimed to purify a lectin from a *Cinachyrella* sp. specimen collected on the northeastern coast of Brazil (CspL) and perform a comprehensive biochemical and structural characterization of the isolated lectin. Although galectins from unidentified *Cinachyrella* species collected in Okinawa, Japan, have previously been reported, the lectin characterized here represents a distinct molecule, as indicated by differences in sequence and biochemical properties. In addition, we evaluated CspL’s antibacterial effects, including its ability to promote bacterial agglutination, modulate antibiotic activity through combinatorial assays, and inhibit biofilm formation.

## 2. Materials and Methods

### 2.1. Animal Collection

Specimens of the marine sponge *Cinachyrella* sp. were collected from the intertidal zone of Pedra Rachada Beach, Paracuru, Ceará, Brazil, under authorization from environmental authorities (SISBIO ID: 33913-12). Samples were transported in plastic bags, stored at −20 °C until use, and identified. A voucher specimen was deposited in the Department of Zoology, Federal University of Pernambuco, under the registration number UFPEPOR4617.

### 2.2. Lectin Isolation

Access to the sponge’s genetic material was authorized by SISGEN (National Genetic Heritage and Associated Traditional Knowledge Management System, ID: A1792FE). After thawing, specimens were cut, homogenized at a 1:2 (*w*/*v*) ratio in 20 mM Tris buffer (pH 7.6) containing 150 mM NaCl (TBS) and 5 mM L-cysteine, filtered through nylon fabric, and centrifuged at 9000× *g* for 30 min at 4 °C. The supernatant (crude extract) was precipitated with 70% ammonium sulfate, centrifuged, and dialyzed.

The resulting fraction (F 0/70) was applied to a DEAE–Sephacel column (2 × 5 cm) equilibrated with 50 mM Tris buffer (pH 7.6). Unbound proteins were removed using the equilibration buffer, whereas bound proteins were eluted with 0.2 M and 0.5 M NaCl in the same buffer. Fractions with hemagglutinating activity were pooled, dialyzed, lyophilized, and further purified by size-exclusion chromatography (SEC) on a Sephacryl S-300 HR column coupled to AKTA Pure (Cytvia, Marlborough, MA, USA). SEC was conducted at 1 mL.min^−1^ using TBS (pH 7.6) containing 5 mM L-cysteine. Active fractions were combined, dialyzed, and lyophilized for subsequent analyses. Protein concentration was determined by the Bradford method [13]. Lectin purification was guided by the evaluation of hemagglutinating activity after each purification step.

### 2.3. Determination of Hemagglutination Activity and Inhibition Assay

Hemagglutination activity was determined by the serial twofold dilution method in V-bottom microtiter plates, following the procedure described by Sampaio, et al. [14] with minor modifications. Rabbit erythrocytes were prepared as 3% suspensions and used either untreated or after trypsinization (CEUAP—Ethics Committee for the Use of Production Animals ID: 2211202101).

For inhibition assays, the lectin solution (4 H.U.mL^−1^) was pre-incubated with various monosaccharides, oligosaccharides, glycoproteins, glycoconjugates or antibiotics for 30 min at room temperature to allow ligand binding. After incubation, an equal volume of the erythrocyte suspension was added to each well, and the plates were gently mixed and left to stand at room temperature for 1 h. The minimum concentration of each inhibitor that completely prevented hemagglutination was recorded as the minimal inhibitory concentration (MIC). The complete list of carbohydrates and glycoproteins tested is presented in Supplementary Table S1.

### 2.4. Effects of pH, Temperature, and Divalent Ions on Hemagglutination Activity

The stability of CspL was evaluated by assessing its hemagglutination activity under different physicochemical conditions, as described by [14], using V-bottom microtiter plates.

To determine pH stability, the lectin was incubated for 1 h in buffer solutions covering a pH range from 4.0 to 10.0. The samples were then equilibrated to room temperature, and residual hemagglutination activity was measured.

Thermal stability was analyzed by incubating the lectin at temperatures ranging from 25 to 100 °C for 60 min, followed by cooling to room temperature before activity determination.

The effect of divalent ions was assessed by incubating the lectin with 10 mM CaCl_2_, MgCl_2_, or MnCl_2_ for 60 min at room temperature prior to hemagglutination testing. To evaluate the role of metal chelation, the lectin was also pre-incubated with 10 mM EDTA under the same conditions.

### 2.5. Estimation of Molecular Mass by SDS-PAGE

The apparent molecular mass, purity, and homogeneity of CspL were analyzed by polyacrylamide gel electrophoresis in the presence of sodium dodecyl sulfate (SDS-PAGE), following the method of Laemmli [15]. The molecular mass was estimated by comparing the migration pattern of the lectin with molecular weight standards from the SigmaMarker™ low-range kit (Sigma Aldrich, St. Louis, MO, USA).

To determine the native molecular mass, the protein was solubilized in TBS containing cysteine and in TBS. Both samples were centrifuged and applied to a BEH SEC 200 Å, 1.7 µm column (4.6 × 300 mm, Waters Corp., Milford, MA, USA) coupled to an Acquity H-Class Bio UPLC system (Waters Corp., Milford, MA, USA). After column equilibration with TBS, the samples were injected and monitored by absorbance at 280 and 216 nm.

### 2.6. Determination of Primary Structure

The amino acid sequence of CspL was determined by tandem mass spectrometry (MS/MS). After separation by SDS-PAGE, the protein bands corresponding to CspL were excised, reduced with dithiothreitol (DTT), and alkylated with iodoacetamide (IAA), as described by Shevchenko, et al. [16]. Proteolytic digestion was performed using trypsin (Promega, Madison, WI, USA), chymotrypsin (Roche, Basel, Switzerland), Glu-C (Roche) and Pepsin (Sigma Aldrich, St. Louis, MO, USA). The protein bands were rehydrated in 25 mM ammonium bicarbonate at a 1:50 (*w*/*w*) enzyme-to-substrate ratio and incubated at 37 °C for 16 h. Reactions were stopped by adding 2 µL of 5% formic acid.

Peptides were extracted according to [16], separated on a C18 reverse-phase nanocolumn (100 μm × 100 mm) using a nanoAcquity UPLC system (Waters Corp., Milford, MA, USA), and eluted with a linear acetonitrile gradient containing 0.1% formic acid. The eluates were directly infused into a nanoelectrospray ionization source coupled to a hybrid ESI-Q-ToF mass spectrometer (Synapt HDMS, Waters Corp., Milford, MA, USA) operating in positive mode (*m*/*z* 50–3000), with a source temperature of 363 K and a capillary voltage of 3.5 kV.

Collision-induced dissociation (CID) spectra were collected and manually interpreted. The resulting peptide sequences were compared with those in databases (NCBI and UniProt) for homology searches, and the primary structure was reconstructed from the overlapping peptides. Leucine and isoleucine residues were distinguished based on the cleavage specificity of chymotrypsin.

### 2.7. Bioinformatics Analysis

Bioinformatic analyses were performed to predict the structural properties of CspL. Domain architecture was identified using the HHPred server [17]. The theoretical isoelectric point (pI) and molecular weight (MW) were calculated using the ProtParam tool [18].

A three-dimensional model of CspL was generated by Alphafold 2 [19], and the amino acid residues potentially involved in carbohydrate binding were predicted using the COACH metaserver [20]. Model validation was performed through stereochemical and structural quality analyses using the MolProbity, QMEAN, and VoroMQA servers [21,22,23,24]. The highest-quality model was visually inspected using PyMOL v3.1.

Multiple sequence alignments were carried out with Multalin, and the results were graphically represented using ESPript [25]. Phylogenetic analysis was performed in MEGA version X, using the neighbor-joining method with 1000 bootstrap replicates to assess the robustness of the tree topology [26].

### 2.8. Circular Dichroism Analysis

Circular dichroism (CD) spectra were recorded to evaluate the secondary structure and thermal stability of CspL. Measurements were performed on a Jasco J-815 spectropolarimeter (Jasco International Co., Tokyo, Japan) equipped with a Peltier temperature control system.

For secondary structure estimation, spectra were collected at 20 °C using a protein concentration of 0.2 mg.mL^−1^ in 20 mM phosphate buffer (pH 7.0). Data were acquired in a 0.1 cm pathlength quartz cuvette, averaging four accumulations per scan, within a wavelength range of 190–250 nm. The spectra were corrected for the buffer baseline and analyzed using the BestSel web server to estimate the secondary structure content [27].

Thermal denaturation experiments were carried out by monitoring ellipticity at a fixed wavelength of 215 nm while increasing the temperature from 20 °C to 100 °C. The melting temperature (Tm) was determined from the transition midpoint of the thermal unfolding curve obtained through BestSel thermal denaturation analysis [27].

### 2.9. Multi-Angle Dynamic Light Scattering (MADLS) Analysis

MADLS analysis was performed to determine the hydrodynamic behavior and oligomeric state of CspL. The lectin was solubilized in TBS at a final concentration of 14 µM. Prior to measurement, the sample was centrifuged at 10,000× *g* for 20 min at 4 °C to remove any insoluble material or aggregates.

Measurements were conducted at 20 °C using a Zetasizer Ultra Red Label (Malvern Panalytical, Worcestershire, UK). The instrument collected light scattering data at multiple detection angles to improve the resolution of particle size distribution. Each measurement was performed in triplicate, with multiple acquisition scans per replicate to ensure reproducibility. The hydrodynamic diameter and polydispersity index (PDI) were calculated from the intensity autocorrelation function using the general-purpose analysis model provided by the manufacturer.

To compare the experimental hydrodynamic parameters with theoretical predictions, a structural model of CspL was submitted to the HullRad server [28]. The apparent anhydrous radius (R_o_) predicted by HullRad was used as a reference for interpreting the MADLS-derived hydrodynamic radius and to infer the oligomeric state of the protein in solution.

### 2.10. Antibacterial Activity

#### 2.10.1. Microorganisms and Culture Conditions

In this study, *Staphylococcus aureus* ATCC 25923, *Staphylococcus aureus* ATCC 700698 (methicillin-resistant *Staphylococcus aureus*—MRSA), and *Escherichia coli* ATCC 11303 were used. The strains were grown in Tryptic Soy Agar (TSA; Himedia, Mumbai, India) and incubated at 37 °C for 24 h. Isolated colonies were transferred to Tryptic Soy Broth (TSB; Himedia, India) and incubated under the same conditions. Cells were harvested by centrifugation (10 min, 9000× *g*), and the bacterial suspensions were adjusted to 1 × 10^6^ (UFC.mL^−1^).

#### 2.10.2. Bacterial Agglutination Assay

Bacterial agglutination assays were conducted according to [29], with modifications. The strains *E. coli* ATCC 11303, *S. aureus* ATCC 25923, and *S. aureus* ATCC 700698 were grown in Tryptic Soy Broth, harvested by centrifugation (2000× *g*, 10 min, 4 °C), washed three times with TBS (50 mM Tris, 150 mM NaCl, pH 7.6), and fixed overnight in 4% formaldehyde at 4 °C. After washing, the bacterial suspensions were adjusted to 2 × 10^8^ CFU.mL^−1^ by turbidimetry at 620 nm.

Equal volumes (50 µL) of CspL solution (1 mg.mL^−1^) and bacterial suspension were mixed and incubated for 1 h at room temperature. To assess carbohydrate inhibition, bacteria were pre-incubated with α-lactose (100 mM, 30 min) before addition of the lectin. Controls consisted of bacterial suspensions with or without α-lactose. Agglutination was observed under a light microscope.

#### 2.10.3. Activity of Lectin on Planktonic Cell Growth of Microorganisms

The effect of the CspL on planktonic growth was evaluated by a broth microdilution assay in 96-well polystyrene plates according to the standards suggested by the Clinical and Laboratory Standards Institute (CLSI) [30], with some modifications [31]. Briefly, bacterial suspensions were prepared in Tryptic Soy Broth (TSB) and adjusted to approximately 1 × 10^6^ CFU.mL^−1^. Aliquots of 100 µL of the bacterial inoculum were added to 96-well microplates containing 100 µL of CspL previously diluted in sterile 150 mM NaCl to final concentrations ranging from 3.9 to 250 µg.mL^−1^. The plates were incubated at 37 °C for 24 h under aerobic conditions.

The lowest concentration of the compound that inhibits visible growth based on turbidity was considered the minimum inhibitory concentration (MIC). Appropriate controls were included: a growth control (medium plus inoculum), a sterility control (medium only), and a solvent control when applicable.

#### 2.10.4. Lectin Activity Combined with Antibiotics

The evaluation of the effect of CspL combined with antibiotics was performed according to [5].

For this study, combinations of CspL with the antibiotics oxacillin (Oxa) and tetracycline (Tetra) were evaluated against *S. aureus* and *E. coli* strains. For each combination tested, the antibiotic was diluted to 1/2× MIC, 1/4× MIC, 1/8× MIC and 1/16× MIC, in combination with the lectin at a constant concentration of 125 μg.mL^−1^. The plates were then incubated at 37 °C for 24 h; for the determination of the new MIC in each combination, the O.D. was measured at 620 nm using a microplate reader (SpectraMax i3 Multi-Mode Microplate Reader, Molecular Devices LLC, San Jose, CA, USA).

#### 2.10.5. Lectin Effect on Biofilm Formation

The evaluation of the effect of the lectin on biofilm formation was carried out according to the method described by [31], with some modifications. The plates were incubated at 37 °C for 24 h to allow biofilm development, in the absence and presence of the lectin, at concentrations ranging from 3.9 to 250 µg.mL^−1^. After this period, the biofilms were washed with sterile 150 mM NaCl to remove loosely adhered cells.

Biomass quantification was assessed using a colorimetric method. The biofilms were fixed with analytical-grade methanol for 15 min, stained with 1% crystal violet for 5 min, and then washed with sterile water. To dissolve the crystal violet, a 33% (*v*/*v*) acetic acid solution was added, and the optical density was measured at 590 nm using a microplate reader (SpectraMax i3 Multi-Mode Microplate Reader).

For the quantification of viable cells within the biofilm, sterile 150 mM NaCl was added to the wells, and the plates were subjected to an ultrasonic bath (Cristófoli, Campo Mourão, Brazil) for 10 min to release the bacterial cells from the biofilm. Subsequently, serial dilutions of the recovered suspensions were performed and plated on TSA medium, followed by incubation at 37 °C for 24 h. The number of colony-forming units was determined, and the results were expressed as Log CFU.mL^−1^.

#### 2.10.6. Statistical Analysis

All experiments were performed in triplicate and repeated in three independent assays. The statistical analysis of the obtained results was conducted using GraphPad Prism^®^ version 9.0 for Microsoft Windows^®^. Data from all experiments were compared using analysis of variance (ANOVA), followed by a Bonferroni post hoc test. A significance level of *p* < 0.05 was considered in the interpretation of the results.

## 3. Results

### 3.1. Lectin Purification

**Figure 1:**
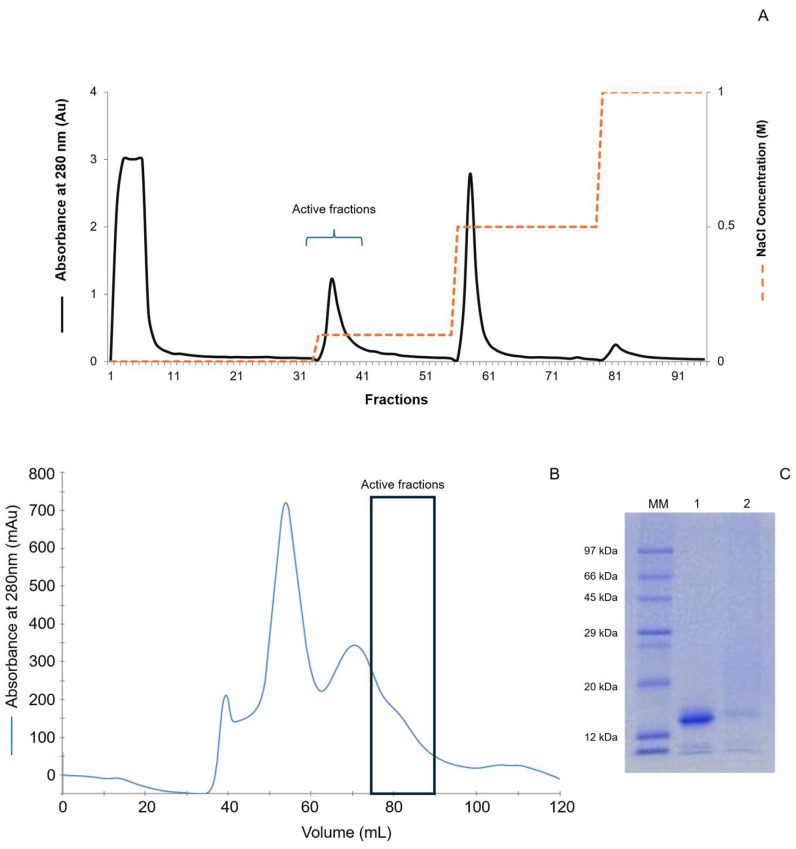
Purification of CspL from the marine sponge *Cinachyrella* sp. (**A**) Ion-exchange chromatography performed on a DEAE–Sephacel column equilibrated with 50 mM Tris-HCl buffer. Bound proteins were eluted stepwise with 0.2 and 0.5 M NaCl. Fractions exhibiting hemagglutinating activity were identified and pooled for subsequent purification. (**B**) Size-exclusion chromatography on a Sephacryl S-300 HR column coupled to an ÄKTA Pure system and equilibrated with TBS containing L-cysteine (pH 7.6). The boxed region indicates the fractions displaying hemagglutinating activity that were pooled for further analyses. (**C**) SDS-PAGE (15%) of purified CspL under non-reducing (1) and reducing conditions (2), revealing a major protein band with an apparent molecular mass of approximately 16 kDa.

The lectin CspL was isolated from the marine sponge *Cinachyrella* sp. and identified in the second peak eluted from the DEAE–Sephacel ion-exchange column with 0.2 M NaCl (Figure 1A). This fraction was lyophilized and further purified by SEC, where CspL was eluted as a broad peak at approximately 80 mL (Figure 1B). Figure 1C shows the SDS-PAGE profile of the purified lectin, which appeared as a single band of approximately 16 kDa, both in the presence and in the absence of β-mercaptoethanol.

### 3.2. Native Molecular Mass and Oligomeric Organization

The oligomeric state of CspL was investigated by SEC and MADLS. In SEC-UPLC, the protein was eluted as a single, symmetric peak corresponding to an apparent molecular mass of approximately 54 kDa (Supplementary Figure S1), suggesting a homotetrameric organization in solution.

**Figure 2:**
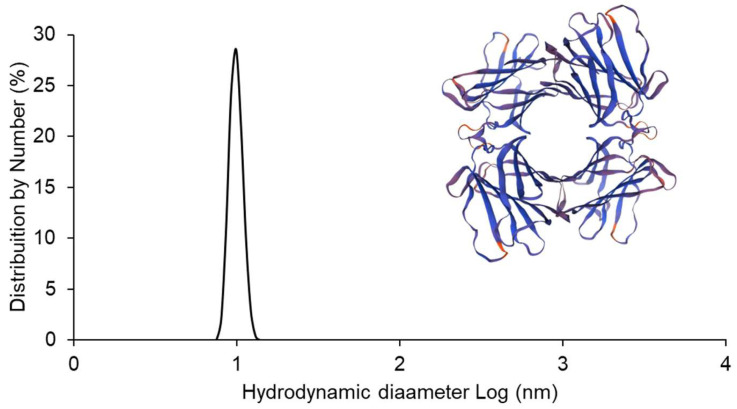
Hydrodynamic diameter distribution of the CspL lectin. Dynamic light scattering (DLS) analysis of CspL (14 µM) revealed a single, sharp peak indicating a population with a hydrodynamic diameter predominantly centered on 5.9 nm, consistent with a homogeneous oligomeric state in solution. The result supports the tetrameric organization predicted by the theoretical three-dimensional model (inset, right), obtained by homology modeling and refined using HullRad, which estimated a maximum dimension of 71.8 Å.

Consistently, MADLS analysis revealed a predominant hydrodynamic diameter of 9.8 nm, with a polydispersity index (PDI) of 0.344 (Figure 2). Although slightly bigger than the dimensions predicted by the tetrameric three-dimensional model obtained through HullRad (Dₘₐₓ = 7.18 nm), the experimental data still support an oligomeric organization compatible with a homotetrameric arrangement of CspL in aqueous solution.

### 3.3. Carbohydrate-Binding Specificity

The hemagglutination activity of CspL was selectively inhibited by a limited set of carbohydrates and glycoproteins (Table 1). The strongest inhibitors were the glycoproteins porcine stomach mucin (PSM) type 3 and type 2, with minimal inhibitory concentrations (MICs) of 0.0078 and 0.0156 mg.mL^−1^, respectively.

Among the monosaccharides and oligosaccharides tested, lactose and lactulose were the most effective inhibitors, with MICs of 3.12 and 6.25 mM, respectively. 4-Phenyl-α-D-galactoside inhibited CspL at 12.5 mM, while 4-phenyl-β-D-galactoside and methyl-β-D-thiogalactoside required higher concentrations (100 and 50 mM, respectively) to suppress lectin activity.

Neither oxacillin nor tetracycline was able to inhibit the hemagglutinating activity of CspL at the tested concentrations, indicating that these antibiotics do not directly compete with the CRD of the lectin. Among the bacterial glycoconjugates evaluated, only lipoteichoic acid (LTA) was capable of inhibiting hemagglutination, whereas lipopolysaccharide (LPS) showed no detectable effect. These findings suggest the preferential interaction of CspL with specific Gram-positive bacterial-surface-associated glycans.

### 3.4. Effects of pH, Temperature, and Divalent Ions on Hemagglutinating Activity

**Figure 3:**
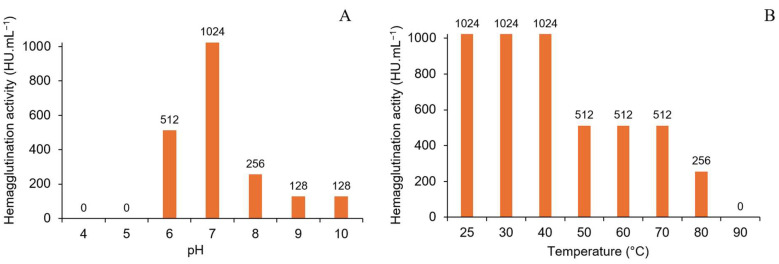
Effects of pH and temperature on the hemagglutinating activity of CspL. (**A**) Influence of pH on the hemagglutinating activity of CspL evaluated after incubation under different pH conditions (pH 4–10). (**B**) Thermal stability of CspL determined after incubation at temperatures ranging from 25 to 90 °C. Hemagglutinating activity is expressed as hemagglutination units per milliliter (HU.mL^−1^).

CspL exhibited maximum hemagglutinating activity at pH 7.0, with approximately 50% loss of activity at pH 6.0 and a marked reduction under alkaline conditions (pH 8–10). Complete loss of activity was accompanied by erythrocyte hemolysis at acidic pH values (4.0–5.0) (Figure 3A).

Thermal stability assays revealed that CspL retained full activity up to 40 °C, followed by a gradual decline, with approximately 50% activity remaining between 50 °C and 70 °C and complete inactivation at 90 °C (Figure 3B). The hemagglutinating activity was not affected by the presence or absence of divalent ions, indicating that CspL is a Ca^2+^-independent lectin.

### 3.5. Primary Structure Determination and Domain Analysis

**Figure 4:**
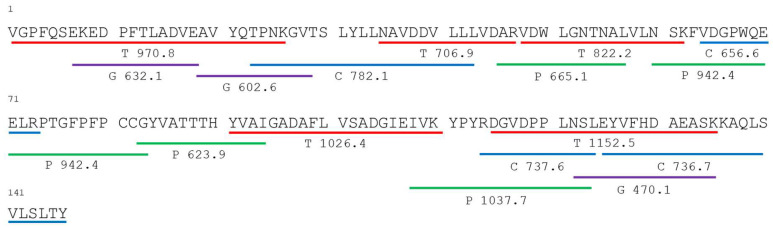
Peptide coverage map of CspL obtained by LC-MS/MS. The amino acid sequence of CspL was deduced from overlapping peptides generated by proteolytic digestions with trypsin (red), chymotrypsin (blue), pepsin (green), and GluC (purple). Together, the identified peptides covered 146 amino acids.

Mass spectrometry analysis of CspL revealed a total of 17 nonredundant peptides obtained from digestions with different proteases: five tryptic, four chymotryptic, three GluC, and five peptic fragments. The overlap of these peptides covered a continuous sequence of 146 amino acids (Figure 4), containing two vicinal cysteine residues, which are likely involved in the formation of an intrachain disulfide bridge. The calculated molecular mass was 16,019 Da, and the theoretical isoelectric point (pI) was 4.31. Additionally, microheterogeneities were detected at several positions, specifically P/S (11), T/F (12), S/T (23), K/T (29), L/V (48), Q/G (83), and N/Y (91), suggesting the presence of subtle sequence variations or isoforms.

Domain prediction using the SMART tool identified a galectin-like domain extending from residues 2 to 146, consistent with the results obtained from HHPred, which showed the highest similarity score for human galectin-3. Conserved amino acids typically involved in carbohydrate recognition among galectins were also detected in CspL, including V28, Y32, V40, V43, D45, R47, W50, V58, N60, K62, and E71, supporting its classification as a member of the galectin family.

**Figure 5:**
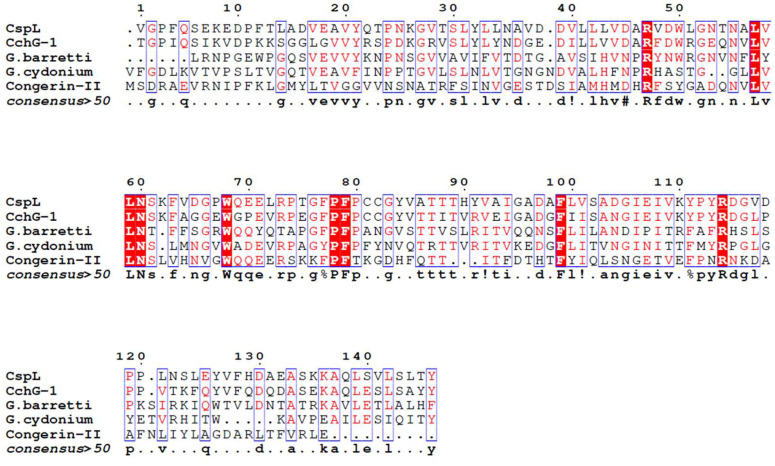
Multiple sequence alignment of CspL with other galectins. The amino acid sequence of CspL was aligned with galectins from *Cinachyrella* sp. (CchG-1, BAM09152.1), *Geodia cydonium* (CAA50198.1), *Geodia barretti* (CAI8019274.1), and congerin from *Conger myriaster* (Q9YIC2.3). Conserved and similar residues are highlighted in red and blue boxes, respectively. The consensus line indicates conserved positions (>50% identity).

Multiple sequence alignment of CspL with other known galectins revealed a high degree of conservation of residues associated with carbohydrate recognition (Figure 5). CspL showed the greatest similarity to the galectin CchG-1, from the sponge *Cinachyrella* sp. (BAM09152.1) collected in Okinawa (Japan). CspL and CchG-1 share 60% identity and 71% similarity. It also shared 36% identity and 51% similarity with galectin-2 from *Geodia cydonium* (CAA50198.1) and 33% identity and 53% similarity with the putative galectin from *Geodia barretti* (CAI8019274.1). Lower similarity was observed with the congerin lectin, from the marine eel *Conger myriaster* (Q9YIC2.3), which exhibited 28% identity and 42% similarity.

**Figure 6:**
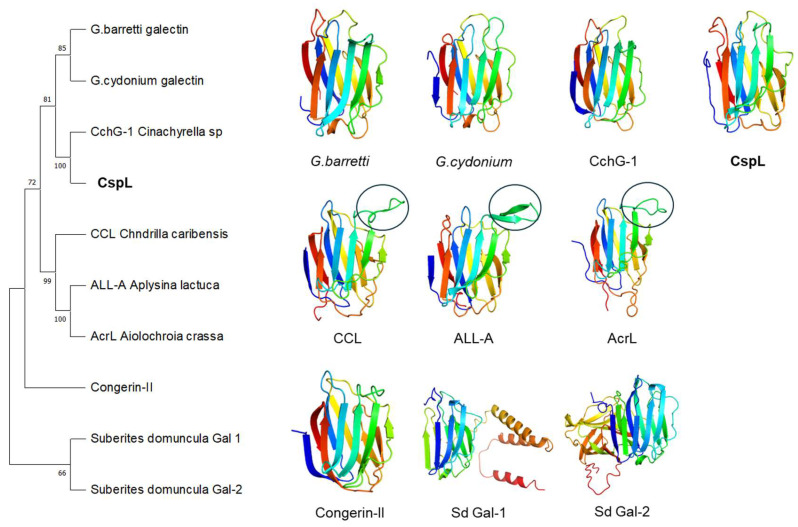
Phylogenetic and structural relationships among sponge galectins and related proto-type galectins. Phylogenetic analysis and comparative structural modeling of CspL and representative galectins from marine sponges and other metazoans. The phylogenetic tree highlights the clustering of sponge galectins into two major subgroups. One subgroup, comprising galectins from *Cinachyrella* sp., *Geodia cydonium*, and *G. barretti*, displays a more canonical proto-type galectin architecture, characterized by the typical jelly-roll β-sandwich fold. In contrast, the second subgroup, including CCL, ALL-A, and AcrL, exhibits additional structural elements adjacent to the carbohydrate-recognition domain (CRD), represented by the extended loop highlighted by circles. These structural differences may reflect distinct glycan-recognition strategies among sponge galectins. Representative structural models are shown alongside the phylogenetic tree for comparative analysis.

The phylogenetic tree constructed using the neighbor-joining method (Figure 6) revealed the clustering of CspL with other sponge galectins into two main groups. In the first cluster, CspL grouped closely with the galectin CchG-1 from *Cinachyrella* sp. and the galectins from *Geodia cydonium* and *G. barretti*, all belonging to the order Tetractinellida. This cluster supports the evolutionary conservation of galectins within this order of demosponges.

The second cluster included the galectins from *A. lactuca* (ALL-A) and *A. crassa* (AcrL), both members of the family Aplysinidae, as well as *C. caribensis*, which belongs to the distinct order Chondrosida. The grouping of these sequences, despite taxonomic divergence, suggests potential functional convergence among galectins from sponges with similar ecological niches or carbohydrate-binding profiles.

The third clade contained Congerin-II, serving as an outgroup to the sponge sequences, while *Suberites domuncula* galectins (Gal-1 and Gal-2), from a sponge of a different order (Suberitida), formed a separate and more basal lineage.

### 3.6. Circular Dichroism

**Figure 7:**
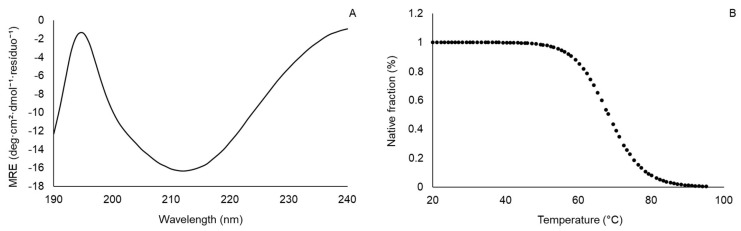
Circular dichroism (CD) analysis of CspL. (**A**) Far-UV CD spectrum of CspL recorded at 20 °C, showing a maximum near 195 nm and a pronounced negative band at 212 nm, characteristic of a protein with a predominantly β-structured conformation. (**B**) Thermal denaturation curve obtained by monitoring ellipticity at 215 nm from 20 °C to 100 °C. The melting temperature (Tm) was calculated as 68.3 °C.

The far-UV CD spectrum of CspL exhibited a maximum band near 195 nm and a pronounced negative band with a minimum at 212 nm (Figure 7A), which are characteristic features of proteins with a predominantly β-structured conformation. The intensity of the 212 nm band further suggests the presence of mixed β-sheets (parallel and antiparallel) and a compact, well-folded conformation in solution. Analysis of the spectra using BestSel revealed that CspL is composed of approximately 11% α-helix, 31% β-sheet, 21% β-turn, and 35% random coil structures.

Thermal denaturation monitored at 215 nm (Figure 7B) demonstrated that the lectin retained its native conformation up to approximately 60 °C, followed by a gradual unfolding transition. The calculated melting temperature (Tm) was 68.3 °C.

### 3.7. Three-Dimensional Model

**Figure 8:**
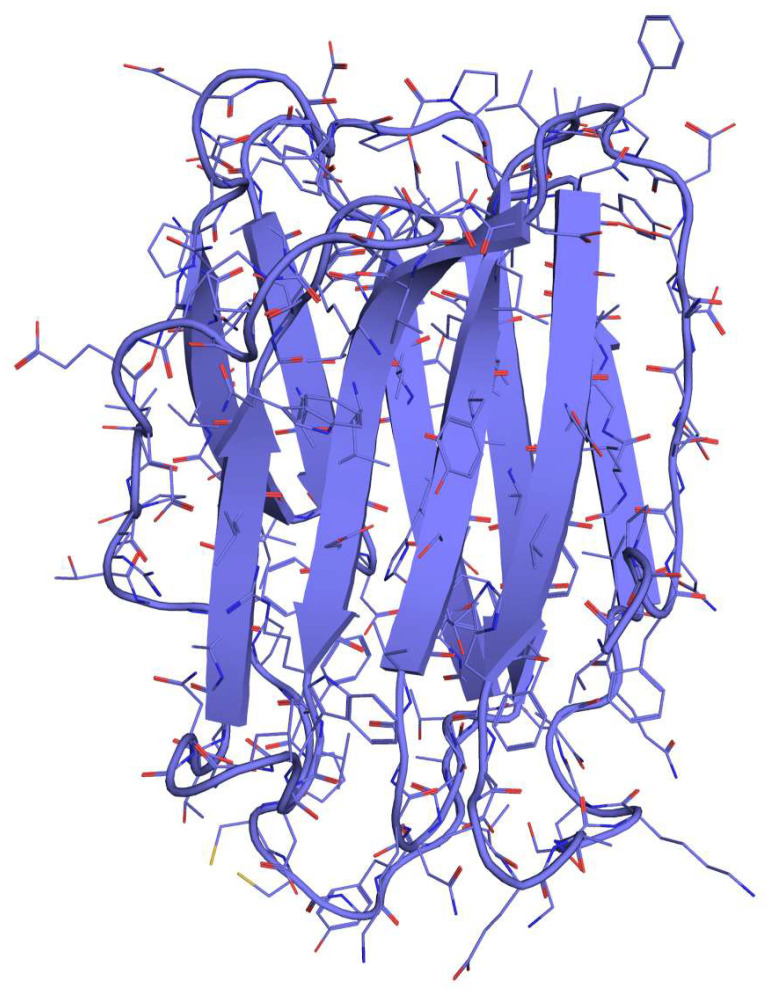
Predicted three-dimensional structure of the CsppL protomer. Ribbon representation of the predicted CsppL structure showing the canonical jelly-roll β-sandwich fold characteristic of galectins. The model is predominantly composed of antiparallel β-sheets surrounding the putative CRD. Side chains are displayed as sticks to highlight the overall organization of surface-exposed residues and the compact structural arrangement of the protomer.

The predicted three-dimensional structure of CspL revealed a typical galectin fold composed of two antiparallel β-sheets arranged in a jelly-roll β-sandwich architecture (Figure 8).

The structural quality of the model was further assessed using complementary validation servers. MolProbity analysis showed that 85.42% of the amino acid residues were located in favored regions of the Ramachandran plot, while 98.39% of side-chain rotamers were classified as favored. In addition, VoroMQA analysis yielded an overall score of 0.441, and QMEAN evaluation resulted in a QMEAN4 value of 0.61. Together, these parameters support the general reliability of the predicted CspL model, although the stereochemical distribution indicates that local regions should be interpreted with caution.

The modeled protomer exhibited a structural organization consistent with that of canonical proto-type galectins, with conserved β-strand topology surrounding the putative CRD. Oligomeric modeling suggested the formation of a tetrameric assembly organized as a “dimer-of-dimers”, generating a toroidal arrangement similar to that previously described for the crystallographically resolved galectin CchG-1 (PDB code: 4agg). The interfaces between protomers appear to involve extensive β-sheet contacts within dimers, whereas tetramer stabilization is likely mediated by loop-mediated interactions between adjacent dimers. Overall, the predicted architecture supports a compact and structurally conserved galectin organization.

### 3.8. Antibacterial Activity

#### 3.8.1. Bacterial Agglutination

The lectin CspL exhibited selective agglutination among the bacterial strains tested. No agglutination was observed for *E. coli* ATCC 11303. In contrast, CspL effectively agglutinated both *S. aureus* strains, including ATCC 25923 and the MRSA strain ATCC 700698, even at concentrations as low as 0.1 mg.mL^−1^ (Supplementary Figure S2).

The addition of 0.1 M lactose partially inhibited the agglutination of *S. aureus* strains, indicating that the interaction between CspL and the bacterial surface is carbohydrate-dependent. The inhibition became more pronounced when lower lectin concentrations were employed, suggesting a specific and saturable binding mechanism.

#### 3.8.2. Antibacterial Activity and Combined Action with Antibiotics

CspL did not completely inhibit bacterial growth under the tested conditions, and, therefore, no minimum inhibitory concentration (MIC) values were determined. However, the lectin promoted significant reductions in bacterial growth, particularly at concentrations up to 125 μg.mL^−1^. Based on this partial inhibitory effect, the concentration of 125 μg.mL^−1^ was selected for subsequent combination assays with antibiotics.

Notable reductions in the individual MIC values of oxacillin and tetracycline were observed when combined with CspL against *S. aureus* ATCC 25923, indicating a synergistic effect for both antibiotics. In contrast, for *S. aureus* ATCC 700698, the combination with CspL resulted in an increased MIC of oxacillin, suggesting an antagonistic interaction. Regarding *E. coli* ATCC 11303, the combination with CspL did not alter the MIC of oxacillin. Tetracycline, however, exhibited a consistent synergistic effect when associated with the lectin across all tested strains (Table 2).

#### 3.8.3. Lectin Effect on Biofilm Formation

**Figure 9:**
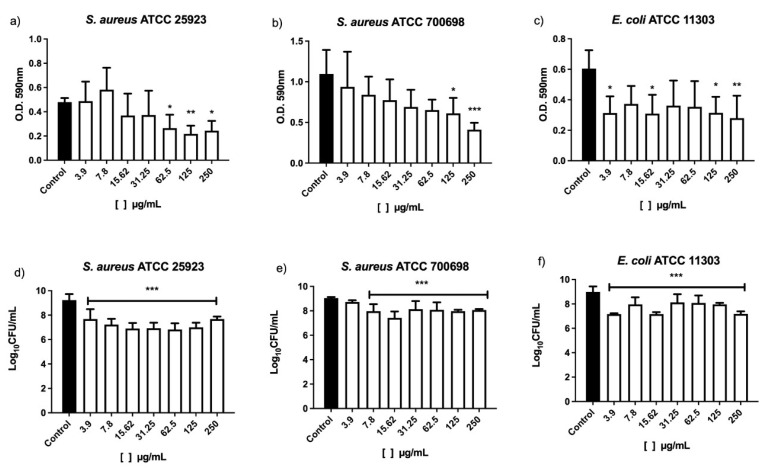
Effect of CspL on biofilm formation of *S*. *aureus* and *E*. *coli* strains. (**a**–**c**): Biofilm biomass quantification; (**d**–**f**): viable cell counts. Black bars represent untreated groups (controls), and white bars represent samples treated with CspL. Statistical significance was determined in comparison to untreated controls using one-way ANOVA followed by Bonferroni post hoc test; *p* < 0.05 (*), *p* < 0.01 (**), *p* < 0.001 (***).

The lectin CspL exhibited significant inhibition of biofilm formation in all bacterial strains tested. In the strain *S*. *aureus* ATCC 25923, a marked, dose-dependent reduction in biofilm biomass was observed, with significant inhibition at concentrations between 62.5 and 250 µg.mL^−1^, leading to an overall 60% decrease (Figure 9a). For the MRSA, CspL also induced a dose-dependent inhibition, with significant effects from 31.25 to 250 µg.mL^−1^ and an approximately 50% reduction in biofilm biomass (Figure 9b). In *E. coli* ATCC 11303, inhibition was evident at 125 and 250 µg.mL^−1^, resulting in a comparable 50% decrease (Figure 9c).

CspL caused a significant reduction in cell viability within the biofilms of *S*. *aureus* ATCC 25923, decreasing viable cell counts by approximately 2.5 log_10_ CFU.mL^−1^ across the same concentration range (Figure 9d). For *S*. *aureus* (MRSA), the reduction reached around 1 log_10_ CFU.mL^−1^ at concentrations between 7.8 and 250 µg.mL^−1^ (Figure 9e). In contrast, for *E*. *coli*, significant decreases in CFU counts were detected only at the highest concentrations (125 and 250 µg.mL^−1^), corresponding to a reduction of approximately 1.5 log_10_ CFU.mL^−1^ (Figure 9f).

## 4. Discussion

In this study, a lectin designated CspL was isolated from the marine sponge *Cinachyrella* sp. through a combination of classical biochemical approaches, yielding a homogeneous protein suitable for structural and functional analyses. The obtained lectin exhibited features consistent with galectin-like proteins, providing a relevant model to explore the relationship between structural organization and biological activity of sponge-derived lectins.

CspL exhibits structural and biochemical characteristics that place it within the group of sponge proto-type galectins. The predominance of β-sheet content, as revealed by circular dichroism, together with sequence similarity and in silico structural predictions, supports this classification. Moreover, the dependence on reducing conditions to maintain carbohydrate-binding activity is consistent with the redox-sensitive behavior commonly reported for galectins [2,32]. This property is typically associated with the presence of cysteine residues susceptible to oxidation, which may lead to nonspecific disulfide bond formation, protein aggregation, and loss of activity [5,7]. Accordingly, the inclusion of reducing agents during purification has been widely adopted to preserve lectin functionality, as previously reported for galectins such as ALL and AcrL [3,5].

CspL displays a quaternary organization consistent with a homotetramer. Although the apparent molecular mass estimated by SEC (~54 kDa) is slightly lower than the theoretical mass expected for a tetramer (~64 kDa), such discrepancies are frequently observed for oligomeric lectins and may arise from differences in hydrodynamic behavior, molecular compactness, hydration state, and interactions with the chromatographic matrix. Notably, the MADLS-derived hydrodynamic diameter (~9.8 nm) exceeded the dimensions predicted by structural modeling; this discrepancy may arise from the combined effects of hydration, conformational flexibility, transient self-association in solution, and sample polydispersity, all of which can influence apparent particle size measurements obtained by light scattering techniques.

A tetrameric state has been reported for CchG-1 from *Cinachyrella* sp. (Okinawa species), as well as for other marine galectins such as ALL, and is characteristic of proto-type galectins, which typically assemble as dimers or tetramers [5,10,11,12]. Considering the monomeric mass of approximately 15–16 kDa, the tetrameric organization of CspL is likely analogous to that described for CchG-1, in which protomers adopt the canonical β-sandwich fold and assemble into a compact “dimer-of-dimers” architecture [8]. This structural arrangement suggests that CspL may share a similar spatial organization of carbohydrate-binding sites, which could have direct implications for its interaction with multivalent glycans and bacterial surfaces. Although both proteins were isolated from unidentified species of the genus *Cinachyrella* and share the overall structural hallmarks of proto-type sponge galectins, several lines of evidence support the classification of CspL as a lectin distinct from the previously described CchG-1. In addition to their distant geographic origins (Brazilian versus Okinawan specimens), the two lectins differ in amino acid sequence, carbohydrate-recognition profile, and biological activities. While CchG-1 was originally identified through its modulatory effects on mammalian glutamate receptors and displays strong recognition of lactose and GalNAc-containing ligands [12], CspL exhibits a distinct inhibition profile characterized by preferential recognition of mucin-derived glycans and differential responses to galactoside derivatives.

Sequence analysis further supports the classification of CspL as a proto-type galectin. The lectin shares significant similarity with galectins from marine sponges, including *Cinachyrella* sp., *Geodia cydonium*, and *G. barretti*, as well as with vertebrate galectins such as that from *Conger myriaster*, indicating a conserved evolutionary framework. Importantly, residues typically involved in carbohydrate recognition were identified, and key motifs associated with the CRD were preserved. In particular, the region corresponding to the conserved motif ^55^NVLVLNSK^62^, previously described in CchG-1, was maintained, with only minor substitutions. Although subtle variations in canonical peptide regions were observed, such as modifications in the heptapeptide (^42^LLVDARV^48^) and positional shifts in short motifs (^77^FPF^79^), similar deviations have been reported for other sponge galectins, including CCL, ALL, and AcrL [7]. These findings suggest that, despite sequence-level adaptations, CspL retains the core structural determinants required for galectin function, supporting its classification within this family while highlighting the inherent plasticity of sponge-derived lectins.

Phylogenetic analysis positioned CspL within a well-defined clade comprising galectins from sponges of the order Tetractinellida, supporting the evolutionary conservation of this lectin family within Demospongiae. Notably, the phylogenetic tree also revealed the presence of two distinct subgroups of sponge galectins. The first includes lectins such as ALL, AcrL, and CCL, which are characterized by the presence of additional structural elements, including extended loops or peptide insertions adjacent to the carbohydrate-recognition domain (CRD), and are often associated with enhanced interactions with complex glycans [7]. In contrast, the second subgroup comprises galectins from genera such as *Cinachyrella* and *Geodia*, which retain a more canonical galectin architecture, with conserved CRD organization and fewer structural insertions.

The carbohydrate inhibition profile of CspL provides further insight into its binding preferences and supports its classification within the canonical subgroup of sponge galectins. Interestingly, although galectins are classically described as β-galactoside-binding proteins, CspL exhibited stronger inhibition by 4-phenyl-α-D-galactopyranoside than by the corresponding β-anomer. Similar deviations from the canonical β-galactoside preference have been reported for other galectins, indicating that carbohydrate recognition within this protein family is more diverse than originally appreciated [33,34]. Additionally, the lectin was effectively inhibited by free galactosides, such as lactose, and exhibited strong inhibition in the presence of PSMs, indicating a preference for Gal- and GalNAc-containing structures.

Notably, in contrast to galectins bearing extended structural elements, such as ALL, AcrL, and CCL, which display higher affinity toward complex glycoproteins like fetuin and asialofetuin [3,5,6], CspL appears to preferentially interact with simpler or more exposed carbohydrate motifs. This distinction likely reflects structural differences between these galectin subgroups. Glycoproteins such as fetuin and asialofetuin are enriched in complex LacNAc-containing N-glycans, which may be more effectively accommodated by galectins possessing extended loops adjacent to the CRD, allowing interactions with bulkier glycan scaffolds. In contrast, mucins are predominantly composed of shorter O-linked glycans, including T and Tn antigens, which may be more accessible to canonical galectins such as CspL. The strong inhibition observed with mucins, despite the absence of interactions with more structurally complex glycoproteins, suggests that CspL favors accessible carbohydrate epitopes over extended glycan architectures.

The agglutination of both *S. aureus* strains by CspL suggests that the lectin interacts with conserved carbohydrate determinants present on the staphylococcal surface. Among these structures, wall teichoic acids are particularly relevant because they are abundant surface polymers involved in cell wall architecture, adhesion, and host interaction [35]. WTAs contain glycosyl substituents, especially *N*-acetylglucosamine residues and, in some strains, galactose-containing residues, whose linkage type and abundance vary among strains, which may serve as recognition motifs for CspL [36]. The ability of teichoic acid to inhibit the hemagglutinating activity of the lectin further supports the participation of these glycoconjugates in carbohydrate recognition. Therefore, the interaction of CsppL with *S. aureus* may depend on conserved WTA-associated glycans.

In contrast, Gram-negative bacteria such as *E. coli* possess LPS in the outer membrane, which consists of lipid A, a core oligosaccharide, and the O-antigen polysaccharide [37]. Although LPS structures can contain diverse carbohydrate motifs, including hexoses and N-acetylated sugars, their accessibility and spatial organization may limit their effective recognition by CspL. Thus, differences in the composition, density, and exposure of surface glycoconjugates between strains may explain the selective agglutination observed.

Furthermore, bacterial agglutination was inhibited in the presence of 0.1 M lactose, indicating that the carbohydrate-binding site is directly involved in this activity. The ability to agglutinate bacterial strains has also been observed in other marine lectins. Galectins such as CCL and AcrL were also capable of agglutinating strains of *S*. *aureus*, *S*. *epidermidis*, and *E. coli* [3,38]. ALL was able to agglutinate strains of *S*. *aureus* and *E. coli* [39]. The ability to agglutinate pathogenic bacteria is frequently observed in lectins from marine invertebrates and may be associated with their chemical defense systems [40,41].

CspL was also evaluated in combination with antibiotics from two different classes: oxacillin, a β-lactam that targets penicillin-binding proteins (PBPs) involved in cell wall synthesis [42], and tetracycline, which inhibits protein synthesis by binding to the 30S ribosomal subunit [43]. The bacterial strains used in this study exhibit distinct susceptibility profiles, including methicillin-resistant *S. aureus* ATCC 700698 (MRSA), which displays reduced susceptibility to β-lactam antibiotics due to the expression of altered PBPs. Under these conditions, CspL enhanced the activity of oxacillin against the susceptible *S. aureus* ATCC 25923 strain, whereas an antagonistic effect was observed in the MRSA strain, suggesting that the outcome of the interaction depends on the bacterial cell wall organization and resistance phenotype. Although the mechanism underlying this phenomenon remains unclear, these opposite effects may be related to differences in cell wall architecture and β-lactam resistance mechanisms between these strains. In MRSA, the presence of PBP2a, a penicillin-binding protein with low affinity for β-lactam antibiotics, may limit the beneficial effects of lectin-mediated surface interactions. Additionally, the bacterial agglutination promoted by CspL could affect accessibility to the antibiotic’s targets. In contrast, the combination of CspL with tetracycline resulted in a consistent synergistic effect against all tested strains, including *S. aureus* and *E. coli*. Although the mechanism responsible for this effect remains unclear, the ability of CspL to recognize bacterial surface glycoconjugates and promote cell agglutination suggests that lectin–bacteria interactions may influence bacterial susceptibility to tetracycline. Nevertheless, the molecular basis of this interaction remains to be elucidated and should be addressed in future studies.

Synergistic or additive effects resulting from the combination of antibiotics with galectins from marine sponges have been previously reported. Lectins such as ALLs and AcrL have demonstrated synergistic and additive effects when combined with antibiotics against pathogenic bacterial strains [3,5].

CspL inhibited biofilm formation in both *S. aureus* strains and in *E. coli*, with a more pronounced effect on biofilm biomass. This reduction is particularly relevant, as biofilm biomass largely reflects the extracellular polymeric substance (EPS) matrix, a key structural component responsible for biofilm integrity and persistence [44]. The EPS matrix acts as a physical and chemical barrier, limiting antimicrobial penetration, sequestering antimicrobial agents, and contributing to the establishment of physiological gradients that promote bacterial tolerance and persistence [45]. Therefore, targeting biofilm biomass may represent an effective strategy to disrupt biofilm architecture and enhance susceptibility to antimicrobial agents. Additionally, the lectin reduced cell viability in all three strains, with greater efficacy observed against *S. aureus*. The mechanism underlying the antibiofilm activity of CspL remains to be fully elucidated. Previous studies with the marine sponge proto-galectin AcrL demonstrated marked alterations in biofilm organization and bacterial membrane integrity, as evidenced by SEM and CLSM analyses [3]. Considering the structural similarities between these lectins and their comparable antibiofilm properties, future microscopy studies may help determine whether similar structural effects also occur in CspL-treated biofilms. In addition, the ability of CspL to agglutinate bacterial cells, together with the inhibition of this activity by fetuin, suggests that interactions with surface glycoconjugates may contribute to its biological effects. Furthermore, marine sponge lectins are known to recognize microbial glycoconjugates and pathogen-associated molecular patterns (PAMPs) [46,47], which has been proposed as an important feature of their antimicrobial activity.

Despite the promising antimicrobial and antibiofilm activities exhibited by CspL, its potential therapeutic application cannot be fully assessed without evaluating its safety profile. Since lectins are capable of interacting with glycoconjugates present on mammalian cells, future studies should investigate the cytotoxicity, hemocompatibility, and overall biocompatibility of CspL. Considering the conservation of key carbohydrate-recognition residues between CspL and vertebrate galectins, including human galectin-3, it is plausible that glycan-mediated host–microbe interactions have undergone functional convergence. Mammalian galectins are known to recognize galactose-containing glycans exposed on microbial surfaces, promoting bacterial recognition, aggregation, and antimicrobial responses. In this context, the ability of CspL to agglutinate bacterial cells and interfere with biofilm development may reflect an ancestral glycan-recognition strategy that emerged early during metazoan evolution and was subsequently conserved in more complex immune systems [48].

## 5. Conclusions

CspL represents a structurally conserved yet functionally distinct sponge galectin, reinforcing the remarkable diversity of lectins within the genus *Cinachyrella*. The integration of biochemical, structural, phylogenetic, and functional analyses supports the existence of different architectural strategies among sponge galectins, which appear to directly influence glycan recognition and biological activity. In particular, the canonical organization observed for CspL contrasts with the extended architectures described for other sponge galectins, suggesting alternative mechanisms for the interaction with glycoconjugates and bacterial surfaces. Together, these findings expand the current understanding of galectin evolution in basal metazoans and highlight marine sponge lectins as promising models for studies involving carbohydrate recognition and antimicrobial applications.

## Figures and Tables

**Table 1:** Inhibition of the hemagglutinating activity of CspL by sugars and glycoproteins.

Sugars	MIC *
Lactose	3.12 mM
Lactulose	6.25 mM
4-Phenyl-α-Galactoside	12.5 mM
β-Methyl-Thiogalactoside	50 mM
4-Phenyl-β-Galactoside	100 mM
Glycoproteins	
PSM type 3	0.0078 mg.mL^−1^
PSM type 2	0.0156 mg.mL^−1^
Bacterial Glycoconjugates	
Lipotechoic acid *S. aureus*	0.5 mg.mL^−1^

* Minimal inhibition concentration.

**Table 2:** Effect of CspL combined with oxacillin and tetracycline on *S. aureus* and *E. coli* strains.

Strains Bacterial	Antibiotic			Effect	Antibiotic			Effect
	Oxa				Tetra			
	MIC^a^ µg.mL^−1^	MIC^b^ µg.mL^−1^	MIC Ratio		MIC^a^ µg.mL^−1^	MIC^b^ µg.mL^−1^	MIC Ratio	
*S. aureus* ATCC 25923	0.250	0.0625	1/4	(S)	0.250	0.0156	1/16	(S)
*S. aureus* ATCC 700698	256	512	2	(AN)	128	32	1/4	(S)
*E. coli* ATCC 11303	256	256	1	(NI)	1	0.250	1/4	(S)

MIC^a^ = MIC value of antibiotic alone; MIC^b^ = novel MIC value of antibiotic combined with CspL. MIC ratios were calculated as MIC combined/MIC alone. Interaction profiles were classified as follows: 1/2, additive effect; ≤1/4, synergistic effect; =1, no interaction; >1, antagonistic effect [5]. S: synergistic; AN: antagonistic; NI: no interactions. Oxa = oxacillin; Tetra = tetracycline.

## Data Availability

The original contributions presented in this study are included in the article/Supplementary Material. Further inquiries can be directed to the corresponding authors.

## Supplementary Materials

The following supporting information can be downloaded at https://www.mdpi.com/article/10.3390/microorganisms14071442/s1: Table S1. Monosaccharides, disaccharides, oligosaccharides, and synthetic glycosides tested for inhibition of CsppL hemagglutinating activity. Figure S1. Estimation of the native molecular mass of CspL by analytical size-exclusion chromatography. Purified CspL was analyzed on a BEH SEC 200 Å column (4.6 × 300 mm, 1.7 μm; Waters Corp.) equilibrated and eluted with Tris-HCl buffer containing 150 mM NaCl and 5 mM L-cysteine (pH 7.6) at a flow rate of 0.2 mL.min^−1^. The inset shows the calibration curve generated using standard proteins: thyroglobulin (TBT, 670 kDa), apoferritin (443 kDa), β-amylase (200 kDa), alcohol dehydrogenase (ADH, 150 kDa), bovine serum albumin (BSA, 66 kDa), carbonic anhydrase (29 kDa), and lysozyme (14.4 kDa). Based on the calibration curve, the apparent native molecular mass of CspL was estimated to be approximately 54 kDa, consistent with a tetrameric organization. Figure S2. Bacterial agglutination assay of CalL at 1 mg.mL^−1^. (A) TBS control with *S. aureus* ATCC 700698; (B–C) *S. aureus* ATCC 700698 incubated with the lectin; (D) *S. aureus* ATCC 700698 incubated with the lectin in the presence of 100 mM lactose; (E) TBS control with *S. aureus* ATCC 25923; (F–G) *S. aureus* ATCC 25923 incubated with the lectin; and (H) *S. aureus* ATCC 25923 incubated with the lectin in the presence of 100 mM lactose.

## Abbreviations

The following abbreviations are used in this manuscript:

ALL	*Aplysina lactuca* lectin
AcrL	*Aiolochroia crassa* lectin
ASF	Asialofetuin
BSM	Bovine submaxillary mucin
CchG-1	*Cinachyrella* sp. galectin (specimens collected in Okinawa, Japan)
CCL	*Chondrilla caribensis* lectin
CD	Circular dichroism
CRD	Carbohydrate-recognition domain
CspL	*Cinachyrella* sp. lectin
DLS	Dynamic light scattering
ITC	Isothermal titration calorimetry
LacNAc	N-acetyllactosamine
LPS	Lipopolysaccharide
LTA	Lipoteichoic acid
MADLS	Multi-angle dynamic light scattering
MIC	Minimum inhibitory concentration
MRSA	Methicillin-resistant *Staphylococcus aureus*
MS/MS	Tandem mass spectrometry
PBS	Phosphate-buffered saline
PDB	Protein Data Bank
PSM	Porcine stomach mucin
SEC	Size-exclusion chromatography
SDS-PAGE	Sodium dodecyl sulfate–polyacrylamide gel electrophoresis
TBS	Tris-buffered saline
T antigen	Galβ1-3GalNAcα1-O-Ser/Thr antigen
Tn antigen	GalNAcα1-O-Ser/Thr antigen
UV	Ultraviolet spectroscopy
