# Supplementary material for: Structural Characterization of a Proto-Type Galectin from Cinachyrella sp. and Evaluation of Its Selective Bacterial Glycan Recognition and Antibiofilm Activity
Source: Microorganisms. 2026 Jun 30;14(7):1442. doi: 10.3390/microorganisms14071442 (PMC13414002; doi:10.3390/microorganisms14071442)
Supplement: Supplementary file 1 [file microorganisms-14-01442-s001.zip › microorganisms-4371349-supplementary.pdf]

Supplementary Table

Table S1. Monosaccharides, disaccharides, oligosaccharides, and synthetic glycosides tested for inhibition of CsppL hemagglutinating activity.

Category	Compound	Full Chemical Name
**Monosaccharides**	D-xylose	D-(+)-Xylose
	D-ribose	D-(+)-Ribose
	L-fucose	L-(−)-Fucose
	L-arabinose	L-(+)-Arabinose
	L-rhamnose	L-(+)-Rhamnose
	D-galactose	D-(+)-Galactose
	D-mannose	D-(+)-Mannose
	D-glucose	D-(+)-Glucose
	D-glucosamine	D-(+)-Glucosamine hydrochloride
	D-galactosamine	D-(+)-Galactosamine hydrochloride
	N-acetyl-D-glucosamine	N-acetyl-D-glucosamine (GlcNAc)
	N-acetyl-D-galactosamine	N-acetyl-D-galactosamine (GalNAc)
	N-acetyl-D-mannosamine	N-acetyl-D-mannosamine (ManNAc)
	D-galacturonic acid	D-(+)-Galacturonic acid
	D-fructose	D-(−)-Fructose
**Disaccharides and Oligosaccharides**	D-sucrose	Sucrose
	D-melibiose	Melibiose
	D-lactose	D-Lactose
	D-lactulose	Lactulose
	D-maltose	Maltose
	D-raffinose	Raffinose
**Synthetic Galactosides and Derivatives**	methyl-α-D-galactopyranoside	Methyl α-D-galactopyranoside
	methyl-β-D-galactopyranoside	Methyl β-D-galactopyranoside
	methyl-β-D-thiogalactoside	Methyl β-D-thiogalactoside
	phenyl-β-D-galactopyranoside	Phenyl β-D-galactopyranoside
	4-nitrophenyl-α-D-galactopyranoside	4-Nitrophenyl α-D-galactopyranoside
	4-nitrophenyl-β-D-galactopyranoside	4-Nitrophenyl β-D-galactopyranoside
	2-nitrophenyl-β-D-galactopyranoside	2-Nitrophenyl β-D-galactopyranoside
**Polysaccharides and glycoproteins**	BSM	Bovine submaxillary mucin
	PSM type-2	Porcine stomach mucin, type II
	PSM type-3	Porcine stomach mucin, type III
	Fetuin	Fetuin from fetal bovine serum
	Asialofetuin	Asialofetuin from fetal calf serum
	Tyroglobulin	Thyroglobulin from porcine thyroid gland
	Mannan	Mannan from *Saccharomyces cerevisiae*

Supplementary Figures

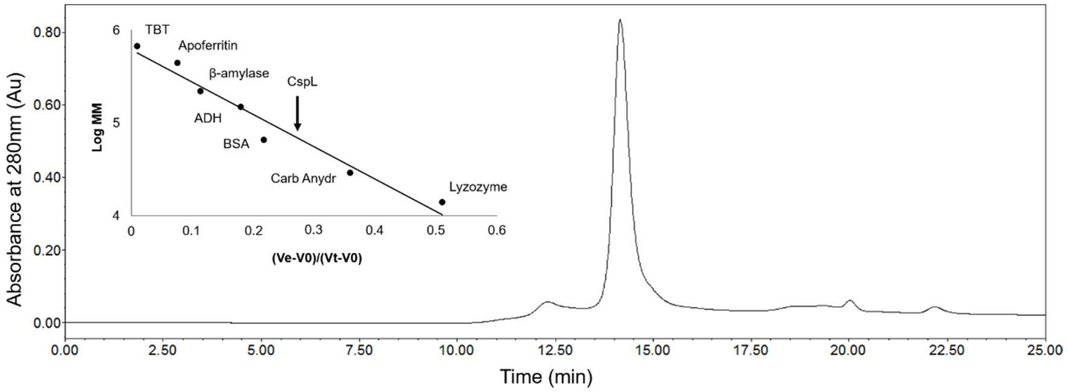

Figure S1. Estimation of the native molecular mass of CspL by analytical size-exclusion chromatography. Purified CspL was analyzed on a BEH SEC 200 Å column (4.6 × 300 mm, 1.7 µm; Waters Corp.) equilibrated and eluted with Tris-HCl buffer containing 150 mM NaCl and 5 mM L-cysteine (pH 7.6) at a flow rate of 0.2mL min^{-1}. The inset shows the calibration curve generated using standard proteins: thyroglobulin (TBT, 670 kDa), apoferritin (443 kDa), β-amylase (200 kDa), alcohol dehydrogenase (ADH, 150 kDa), bovine serum albumin (BSA, 66 kDa), carbonic anhydrase (29 kDa), and lysozyme (14.4 kDa). Based on the calibration curve, the apparent native molecular mass of CspL was estimated to be approximately 54 kDa, consistent with a tetrameric organization.

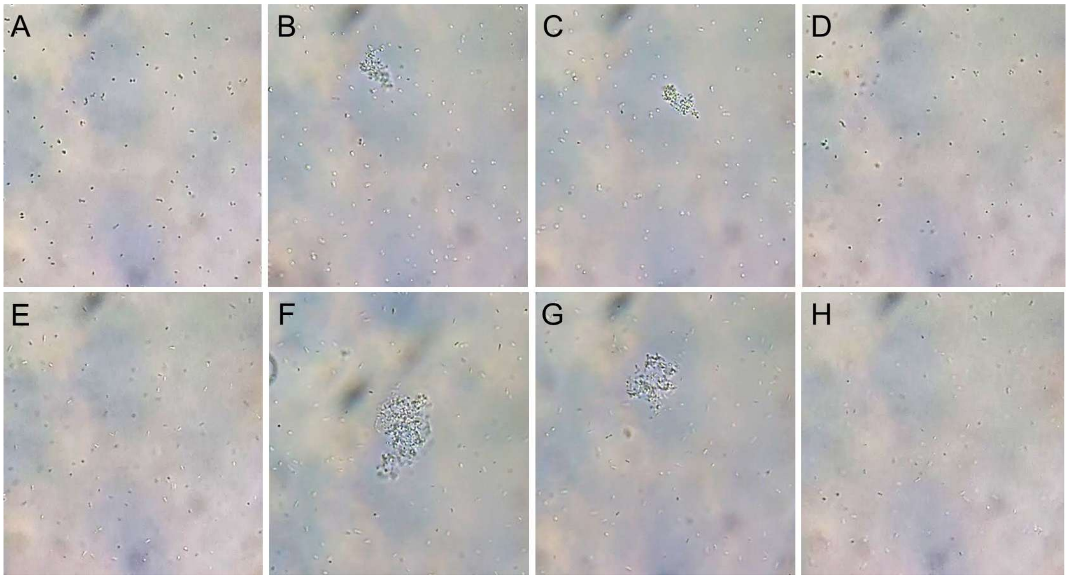

Figure S2. Bacterial agglutination assay of CalL at 1 mg.mL^{-1}. (A) TBS control with *S. aureus* ATCC 700698; (B–C) *S. aureus* ATCC 700698 incubated with the lectin; (D) *S. aureus* ATCC 700698 incubated with the lectin in the presence of 100 mM lactose; (E) TBS control with *S. aureus* ATCC 25923; (F–G) *S. aureus* ATCC 25923 incubated with the lectin; and (H) *S. aureus* ATCC 25923 incubated with the lectin in the presence of 100 mM lactose.
